# Seizures after percutaneous endoscopic lumbar discectomy: A case report

**DOI:** 10.1097/MD.0000000000022470

**Published:** 2020-11-20

**Authors:** Junbei Wu, Yin Fang, Wenjie Jin

**Affiliations:** Department of Anesthesiology, The First Affiliated Hospital of Nanjing Medical University, Nanjing, P.R. China.

**Keywords:** complication, percutaneous endoscopic lumbar discectomy, seizure

## Abstract

**Rationale:**

Percutaneous endoscopic lumbar discectomy (PELD) is a minimally invasive technique for removing nucleus pulposus and achieving neural decompression via a posterolateral approach. PELD is known to have a very low rate of complications during the perioperative period. Although quite rare, seizures can occur in patients undergoing PELD.

**Patient concerns:**

A 58-year-old man with severe low back pain underwent the PELD procedure under general anesthesia. During the recovery phase after general anesthesia, the patient developed a tonic-clonic seizure. Two additional episodes occurred subsequently.

**Diagnoses:**

Bilateral disc swelling indirectly supports the diagnosis of intracranial hypertension.

**Interventions:**

Midazolam and propofol were administered to control seizures. 1.0 g phenobarbital sodium was administered by intravenous injection. Ten milligrams of furosemide and 250 mL of mannitol (20%) were prescribed sequentially. Two hundred milligrams of hydrocortisone and an ice bag were used to protect the brain. Urapidil, metoprolol, and nicardipine were intermittently used to control his blood pressure. A sustained release of sodium valproate was administered and continued prophylactically for 4 weeks.

**Outcomes:**

No further seizures were recorded and the patient recovered well.

**Lessons:**

We conclude that total volume of fluid used for irrigation was considered a possible cause of seizure. This case illustrates the fact that irrigation should be performed cautiously in PELD procedure. And anesthesiologists should be familiar with the management strategies of perioperative acute seizures.

## 1 Introduction

Percutaneous endoscopic lumbar discectomy (PELD) is a minimally invasive technique used to treat lumbar disc herniation, and the operations are performed via a posterolateral transforaminal approach with spinal endoscopy for discectomy and achieving neural decompression. PELD has many benefits, such as less damage to muscular and ligamentous structures, shorter hospital stays, and earlier recovery to function, compared with traditional operations. PELD is also known to be associated with a very low rate of complications during the intraoperative and postoperative periods. Seizures after PELD are quite rare (0.02%) according to previous literature.^[1]^ Perioperative seizures may occur in patients with or without a history of epilepsy. Having a perioperative seizure can lead to circumstances that are dangerous for patients. In severe cases, status epilepticus may develop. The case involves a 58-year-old man who presented with this unexpected rare symptom of seizures accompanied by intractable hypertension immediately after the PELD procedure. Urgent management of these episodes was instituted as soon as possible, and the episodes did not cause any other serious complications. Successful treatment may help to prepare for similar situations in the future.

## 2 Case presentation

### 2.1 Medical history

We report a 58-year-old man with severe low back pain who visited our center for PELD. No other comorbidities were known. No history of epilepsy or neurological disease was reported, and the family history was unremarkable. Preoperative laboratory investigation results and vital signs were within normal limits. Magnetic resonance imaging (MRI) showed protruded lumbar disc (L2/L3, L3/L4) herniation and extruded disc herniation at the L4/L5 level on the right side and at L5/S1 on both sides, leading to dural sac compression. Right L4/5 intervertebral foramen stenosis and bilateral L5/S1 foramen stenosis were observed.

### 2.2 PELD

Standard general anesthesia care was provided, including direct intraarterial pressure monitoring. Anesthesia was induced with 2 mg midazolam IV, 20 mg etomidate IV, and 0.5 mg fentanyl IV infusion followed by 15 mg cis-atracurium IV to facilitate endotracheal intubation. Anesthesia was maintained using 1.7% to 2.5% sevoflurane (1.0–1.5 minimum alveolar anesthetic concentration) with propofol (150 mg/h), remifentanil (0.05–0.2 μg/kg/min), and dexmedetomidine (0.4 μg/kg/min). The patient was placed in the prone position. A fluoroscopic-guided posterolateral transforaminal approach was performed. Afterwards, a working channel endoscope was inserted. Herniated disc fragments (L4/L5) were removed using endoscopic forceps under endoscopic vision. Finally, anatomical details of the epidural and foraminal area, including the decompressed nerve root and dural sac, were confirmed. The surgery lasted 145 minutes, and the irrigation time was 90 minutes, with the continuous infusion of 4000 mL saline into the epidural space. At the beginning of the operation, the irrigation speed was 20 mL/min. When conspicuous endoscopic bleeding occurred during the procedure, the speed increased to 50 to 100 mL/min.

### 2.3 Seizure

The patient's arterial blood pressure (Bp) and heart rate (HR) remained stable during the procedure. The systolic pressure fluctuated between 120 and 140 mmHg. The diastolic pressure fluctuated between 60 and 80 mmHg. During the recovery phase after general anesthesia (25 minutes after the completion of PELD), the patient experienced the sudden onset of seizures with a subsequent sustained high Bp between 180/80 and 248/137 mmHg and an HR of 90 to 118 beats/min. The patient was in the supine position, with the head of his bed up in a 30° angle. The initial seizure was with a presentation of tonic-clonic seizure activity. The second seizure was accompanied by facial tics. When undergoing the computed tomography (CT) examination, the patient opened his eyes unconsciously and experienced a third tonic-clonic seizure. The CT scan showed a lacunar cerebral infarction in the right frontal lobe. No abnormalities in cardiac function were found by bedside transthoracic echocardiography. Blood gas analysis was taken to check for signs of abnormal blood sugar and oxygenation levels and electrolyte imbalances. No abnormality is found. The fundus examination revealed bilateral disc swelling. Emergency supportive treatment was provided immediately. Midazolam and propofol were administered to control the initial postoperative seizures and were administered again because of subsequent repeated seizures. 1.0 g phenobarbital sodium was administered by intravenous injection subsequently. Ten milligrams of furosemide and 250 mL of mannitol (20%) were prescribed sequentially. Two hundred milligrams of hydrocortisone and an ice bag were used to protect the brain. The patient had a significant diuretic response with a total urine output of 2500 mL in the subsequent 4 hours. Urapidil, metoprolol, and nicardipine were intermittently used to control his blood pressure.

Then, the patient was transferred to the intensive care unit (ICU). The axillary temperature was recorded every hour and fluctuated between 36.6 and 37.2 °C. He received remifentanil (0.05–0.2 μg/kg/min) and propofol (150 mg/h) for analgesia and sedation in the ICU. Nicardipine was administered by a continuous infusion at a rate of 0.5 μg/kg/min. The therapy effectively reduced the arterial blood pressure by 10% per hour. The patient was extubated the next morning and denied having a headache, blurred vision, nausea, and other symptoms. Electroencephalography (EEG) was performed during ICU stay. No epileptiform discharge was recorded. No cerebral or subarachnoid hemorrhage or cortical infarction was ever found 48 hours later on CT scan. A sustained release of sodium valproate was administered and continued prophylactically for 4 weeks. No further seizures were recorded.

## 3 Discussion

Although PELD is safe and effective, it has some complications, such as neural and vascular structure injuries, dural tears, infection, and reherniation. Seizures after PELD are quite rare (0.02%) according to previous literatures. Shin et al^[2]^ reported a case of an agnogenic nonepileptic seizure that subsequently developed into Takotsubo cardiomyopathy. Kertmen et al^[3]^ reported a case of an iohexol-induced seizure after transforaminal PELD. Zhou et al^[4]^ reported a case of an agnogenic intraoperative seizure in a 26-year-old man with symptoms of constricted feelings of saddle distribution.

A postoperative seizure is a brain disorder that occurs most commonly in patients undergoing neurosurgery and individuals with epilepsy or pre-existing, undiagnosed brain diseases. Postoperative seizures are rare in patients who have no prior history of seizures before surgery. However, acute incipient postoperative seizures may be associated with metabolic and endocrine disturbances or a wide range of drugs, such as anesthetics, psychotropic drugs, and some antibiotics.^[5]^ The postoperative homeostatic imbalance, which involves hypoxia, hypoglycemia, or hyponatremia, lowers the seizure threshold. We checked blood gas analysis immediately. No abnormality is found. Although anesthetics induced seizures are rare, and underlying mechanisms remain uncertain, anesthetics (propofol, ketamine, sevoflurane, opioids) may be occasionally proconvulsant.^[6]^ All anesthetic drugs used in our operation are easily metabolized without cumulative effects. There was no clear evidence that the series of seizures were related to the anesthetics, unless it was a rare cause of heterogeneity.

Dural tears, rootlet exposure, and cerebrospinal fluid (CSF) leakage may occur during PELD. Unlike in open discectomy, it is comparatively difficult to detect a dural tear during a minimally invasive operation. Musser et al^[7]^ suggested a close temporal relationship between cerebrospinal fluid (CSF) leakage and seizures during cochlear implant surgery. Matsuhiro et al^[8]^ reported a case of a seizure suspected to be subarachnoid hemorrhage due to intracranial hypotension after spinal surgery, which was illustrated by the disappearance of the cerebrospinal fluid cavity on a CT scan. However, the underlying mechanism remains unclear, and the literature on intracranial hypotension as a cause of seizure is limited to case reports.^[9–11]^

In our case, a CT scan showed a lacunar cerebral infarction in the right frontal lobe, which may be an explanation, but it is highly unlikely to be the cause of the seizures. The occurrence of early seizures complicating acute stroke is well recognized and is seemingly more common with cerebral or subarachnoid hemorrhage and cortical infarction.^[12,13]^ Among the presentations of lacunar infarcts, seizures have rarely been reported,^[14,15]^ and the relationship is uncertain.^[16]^ Lacunar lesions caused by occlusion of the perforating artery can lead to hyperaemia in the surrounding area. Epileptic activity may be the result of impaired membrane potential in this area.^[14]^ However, seizures are more likely the expression of an underlying neurodegenerative process and cause more severe cognitive impairment than lacunar infarcts.^[16,17]^

Irrigation with normal saline is performed to maintain a clear endoscopic visual field, and the speed of irrigation depends on visual accessibility. However, intracranial pressure (ICP) increases when an epidural injection occurs.^[18]^ The continuous infusion of normal saline into the epidural space can compress the thecal sac, which in turn can increase cerebrospinal fluid pressure in a cephalad direction. In a study by Joh et al,^[19]^ the basal irrigation speed was 150 to 300 mL/min, thrice the speed of our irrigation, but the irrigation duration was shorter than ours. Therefore, a long irrigation time and a large total volume, similar to those used in our case study, may lead to fluid retention compared with a high speed. However, available data are limited for defining a time or total volume threshold beyond which a seizure is likely to occur. The diagnosis of intracranial hypertension can usually be confirmed by demonstrating increased CSF pressure after performing lumbar puncture. Although we did not measure the pressure in the subarachnoid space, bilateral disc swelling indirectly supports the diagnosis of intracranial hypertension.

An increase in pressure can restrict blood supply to the brain. This condition may be an emergency. We ascribe the intractable blood hypertension to the rapidly increased ICP in this case, namely, the Cushing response. Additionally, as seizures occurred, a stress response was triggered, which worsened the patient's state. Because of the possible increase in ICP, we immediately administered a dehydrator and glucocorticoid to protect the brain. But there are other points to be considered besides this. Posterior reversible encephalopathy syndrome (PRES) should be also considered in patients who present with seizures, altered consciousness, visual disturbance, or headache, particularly in the context of acute hypertension.^[20]^ Three main mechanisms of PRES included breakthrough theory, vasospasm theory, and endothelial dysfunction.^[21]^ The most widely accepted is the first breakthrough theory. It proposes uncontrolled hypertension causes interruption to brain autoregulation. Typical imaging findings include vasogenic edema within the occipital and parietal region, perhaps relating to the posterior cerebral artery supply.^[22]^ This explanation is debatable in our case. Hypertension is not the trigger but the subsequent concomitant symptoms and radiologic edema was not found. What we should note here is that intractable hypertension can make matters worse through this hyperperfusion mechanism.

To avoid this complication, meticulous preoperative planning is necessary. First, prevention is always better than a cure. Careful manipulation during PELD is always the key to preventing bleeding and dural tears. Pay attention to prodromal symptoms, such as neck pain,^[1,19]^ headaches, blurred vision, and drowsiness. Second, control the irrigation system at a comparatively lower pressure. The irrigation pressure is usually controlled at 25 to 30 mmHg.^[19]^ The formula known as Poiseuille law states that the flow of a liquid (*Q*) depends on following factors like the pressure gradient (Δ*P*) at the entrance and outlet of the tube, the length (*l*) and radius (*r*) of it, and the viscosity of the fluid (*η*). The entire relation is given by, *Q* = *π* Δ*P r*^4^/8*ηl*. The pressure and flow are related to each other. Because the pressure was not routinely monitored during PELD, monitoring the irrigation speed was more practical. No previous literature has mentioned the speed threshold value, which we consider to be lower than at least 150 mL/min. Third, shorten the duration of the procedure, especially the irrigation duration. Sustained compression of the epidural space, even with a relatively low and steady irrigation speed, may increase the pressure. Last, we should keep in mind that anesthesia itself may cause seizures during the perioperative period. Regardless of the reason, anesthesiologists should be familiar with the management strategies of perioperative seizures.

The diagnosis has some limitations. First, an epidural catheter was not inserted during PELD. Theoretically speaking, an epidural catheter can be attached to a disposable pressure transducer that was connected to a monitoring system. Thus, the epidural pressure is available. Without this pressure system, we did not have a sufficient amount of evidence that the irrigation pressure was exceedingly high. Second, the diagnosis was limited because there were no electroencephalogram (EEG) records during the seizures. The EEG signals may reveal a pattern and indicate whether a seizure is likely to occur again. The signals may also help to exclude other conditions that mimic epilepsy as a reason for the seizures. Third, cranial MRI, which is more sensitive than CT in detecting epileptogenic lesions, was not available.

## 4 Conclusion

In this case report, we described a patient who developed tonic-clonic seizures during the recovery phase after general anesthesia after the PELD procedure. Normal saline irrigation and seizures suggest a possible causal relationship that is supported by bilateral disc swelling if other causes of seizures are eliminated. Although quite rare, this case serves as a very important reminder for spine surgeons and anesthesiologists of this potentially serious complication. Irrigation should be performed cautiously in PELD procedure, by controlling the flow rate and total irrigation time. Regardless of the cause, anesthesiologists should be familiar with the management strategies of perioperative acute seizures.

## Author contributions

**Authorship:** Junbei Wu drafted the manuscript. Yin Fang was involved in critical revision of the manuscript; Wenjie Jin was involved in final approval of the manuscript.

**Conceptualization:** Wenjie Jin.

**Data curation:** Junbei Wu, Yin Fang.

**Formal analysis:** Junbei Wu, Yin Fang.

**Methodology:** Wenjie Jin.

**Supervision:** Wenjie Jin.

**Validation:** Wenjie Jin.

**Visualization:** Wenjie Jin.

**Writing – original draft:** Junbei Wu.

**Writing – review & editing:** Yin Fang, Wenjie Jin.
